# Sensing of Porcine Reproductive and Respiratory Syndrome Virus-Infected Macrophages by Plasmacytoid Dendritic Cells

**DOI:** 10.3389/fmicb.2016.00771

**Published:** 2016-06-02

**Authors:** Obdulio García-Nicolás, Gaël Auray, Carmen A. Sautter, Julie C. F. Rappe, Kenneth C. McCullough, Nicolas Ruggli, Artur Summerfield

**Affiliations:** ^1^The Institute of Virology and Immunology (IVI)Mittelhäusern, Switzerland; ^2^Department of Infectious Diseases and Pathobiology, Vetsuisse Faculty, University of BernBern, Switzerland

**Keywords:** PRRSV, macrophages, plasmacytoid dendritic cells, intercellular communication, IFN-α

## Abstract

Porcine reproductive and respiratory syndrome virus (PRRSV) represents a macrophage (MØ)-tropic virus which is unable to induce interferon (IFN) type I in its target cells. Nevertheless, infected pigs show a short but prominent systemic IFN alpha (IFN-α) response. A possible explanation for this discrepancy is the ability of plasmacytoid dendritic cells (pDC) to produce IFN-α in response to free PRRSV virions, independent of infection. Here, we show that the highly pathogenic PRRSV genotype 1 strain Lena is unique in not inducing IFN-α production in pDC, contrasting with systemic IFN-α responses found in infected pigs. We also demonstrate efficient pDC stimulation by PRRSV Lena-infected MØ, resulting in a higher IFN-α production than direct stimulation of pDC by PRRSV virions. This response was strain-independent, required integrin-mediated intercellular contact, intact actin filaments in the MØ and was partially inhibited by an inhibitor of neutral sphingomyelinase. Although infected MØ-derived exosomes stimulated pDC, an efficient delivery of the stimulatory component was dependent on a tight contact between pDC and the infected cells. In conclusion, with this mechanism the immune system can efficiently sense PRRSV, resulting in production of considerable quantities of IFN-α. This is adding complexity to the immunopathogenesis of PRRSV infections, as IFN-α should alert the immune system and initiate the induction of adaptive immune responses, a process known to be inefficient during infection of pigs.

## Introduction

Porcine reproductive and respiratory syndrome virus (PRRSV) belongs to the genus *Arterivirus* within the family *Arteriviridae*, order *Nidovirales*, and is divided into genotype 1 (PRRSV-1) and genotype 2 (PRRSV-2; Nelsen et al., 1999). Both genotypes present a high degree of inter- and intra-genotype variability with different subtypes varying in virulence. Originally, more virulent isolates were discovered only in genotype 2 which were termed “highly pathogenic” (HP)-PRRSV strains, but recently highly virulent PRRSV-1 isolates were found also in subtype 3 viruses from Eastern Europe (Stadejek et al., 2008; Karniychuk et al., 2010; Shi et al., 2010; Stadejek et al., 2013). The disease syndrome caused by PRRSV is characterized by reproductive failure in sows as well as respiratory disease in piglets and growing pigs (Done and Paton, 1995). The virus has a high prevalence in intensive pig farms causing major economic losses to the swine industry worldwide, with estimated losses of up to $664 million annually in the USA pork industry for instance (Holtkamp et al., 2013).

PRRSV shows a restricted tropism for cells from the monocyte/macrophage lineage, with main target cells for viral replication being the alveolar and other tissue macrophages (MØ; Van Breedam et al., 2010). It is believed that MØ-targeting by PRRSV is associated with the ability of the virus to suppress immune responses resulting in delayed innate and adaptive immune responses and secondary bacterial infections (Solano et al., 1997; Meier et al., 2003; Lopez and Osorio, 2004; Thanawongnuwech et al., 2004; Yoo et al., 2010). Not surprisingly, interferon type I (IFN-I) responses in several monocytic cell populations including porcine alveolar MØ (PAM), monocyte derived dendritic cells (MoDC) or monocyte-derived MØ are suppressed by the virus and several viral genes, and their mechanism of action have been described (Albina et al., 1998; Buddaert et al., 1998; Lee et al., 2004; Chen et al., 2010; Patel et al., 2010; Zhang et al., 2012). Despite this fact, in PRRSV infected pigs systemic IFN alpha (IFN-α) responses have been reported (Van Reeth et al., 2002), which show particularly high levels during highly pathogenic (HP)-PRRSV infection (Guo et al., 2013). A possible source of this IFN-α could be from plasmacytoid DC (pDC), which are able to produce IFN-α after stimulation with PRRSV *in vitro*, although some strain-dependent differences were found (Baumann et al., 2013).

The pDC are a subset of the DC family specialized in sensing nucleic acids, resulting in the production of high levels of IFN-α secretion. Typically, this is induced by triggering endosomal Toll-like receptor (TLR) 7 and TLR9 through exposure to ssRNA or DNA, respectively. These cells produce up to 1000 times more IFN-α than any other cellular type, acting as main source of systemic IFN-I at the early stage of many virus infections (Liu, 2005). In pigs, pDC are well described and represent 0.1–0.3% of blood leukocytes and respond to many porcine viral pathogens that control IFN-I in other cell types. It is evident that the quantity of IFN-α secreted by porcine pDC is highly variable depending on the virus studied. For example, influenza virus and transmissible gastroenteritis virus are amongst the most potent stimulators of pDC while responses induced by porcine circovirus and foot-and-mouth disease virus are rather low in comparison. In this range of IFN-α responses induced by various viruses, PRRSV can be classified as an intermediate simulator of pDC (Baumann et al., 2013). This activation involves TLR7 and is not associated with pDC infection.

Here, we demonstrate that the recently emerged more virulent PRRSV strain Lena is unable to activate pDC *in vitro* while inducing high levels of IFN-α *in vivo*. We found a possible explanation for this discrepancy by demonstrating that PRRSV infected MØ are most potent at activating pDC independently of the strain employed. This activation requires cell-adhesion molecule-mediated contact between MØ and pDC, an intact cytoskeleton and sphingomyelinase activity in the infected MØ but is not caused by free PRRSV virions released from the infected MØ. Thus, pDC function as most efficient sensors of PRRSV-infected cells as described for other viruses.

## Materials and Methods

### Cell Preparations

Monocyte-derived MØ were generated from monocytes, and blood-derived pDC were enriched as previously described (Carrasco et al., 2001; Guzylack-Piriou et al., 2006). Briefly, peripheral blood mononuclear cells (PBMCs) were taken from 6-weeks-old to 12-months-old specific pathogen free (SPF) Swiss Large White pigs (blood sampling approved by the cantonal ethical committee for animal experiments, license #BE88/14) using ficoll-paque density centrifugation (1.077 g/L; Amersham Pharmacia Biotech, Dübendorf, Switzerland). Then, cells were sorted using the CD172a^+^ monoclonal antibody (mAb, clone 74-22-15A, hybridomas kindly provided by Dr. A. Saalmüller, Veterinary University of Vienna, Austria) using MACS with LS columns (Miltenyi Biotec GmbH, Bergisch Gladbach, Germany) for monocytes and LD columns (Miltenyi) for pDC enrichment. This sorting enriched pDC by a factor of 10–20 resulting in a frequency of 2–8% (Baumann et al., 2013). In order to generate MØ, monocytes were seeded in 24-well culture plates at 5 × 10^5^ cells/ml in Dulbecco’s modified Eagle’s medium containing Glutamax (DMEM, Life Technologies, Zug, Switzerland) supplemented with 10% of heat-inactivated SPF porcine serum (produced in-house) and cultured for 3 days at 39°C and 5% CO_2_. Enriched pDC were cultured in DMEM with 10% fetal bovine serum (FBS; Biowest, Nuaillé, France) and 20 μM of β-mercaptoethanol (Invitrogen, Zug, Switzerland). MARC-145 cells (African green monkey kidney derived cells; ATCC, LGC Standards, Molsheim, France) were cultured in 24-well culture plates at 8 × 10^4^ cells/ml in DMEM with 10% of FBS at 37°C and 5% CO_2_. Exosomes released from infected MØ were isolated with the “total exosome isolation reagent” (Life Technologies) according to the manufacturer instructions.

### Viruses

The following PRRSV strains were employed: LVP23, representing Lelystad virus adapted to grow in MARC-145 cells; Lena strain, representing a subtype 3 PRRSV-1 (kindly provided by Prof. Hans Nauwynk, Ghent University, Belgium); VR-2332 (ATCC) a prototype PRRSV-2 strain (Collins et al., 1992), and RVB-581 (kindly obtained from Prof. Martin Beer, Friedrich-Loeffler-Institut, Greifswald - Insel Riems, Germany) representing a HP-PRRSV field strain isolated in China (Wernike et al., 2012). All PRRSV strains used in this study were propagated in MØ cultures as described (Garcia-Nicolas et al., 2014). Virus titers were determined in MØ using the immunoperoxidase monolayer assay (IPMA) with the anti-nucleocapsid (N) mAb SDOW17-A (Rural Technology, Inc., Brookings, SD, USA). Titers were calculated and expressed as 50% tissue culture infective dose per ml (TCID_50_/ml).

### PRRSV Lena-Infection of Pigs

With the purpose of producing sera from pigs infected with a prototype genotype 1 subtype 3 virus for the implementation of diagnostic ELISAs and RT-qPCR for detection of PRRSV-1 subtype 3 infection, three 6-weeks-old SPF pigs were inoculated intranasally with 5 ml of DMEM containing 5 × 10^5^ TCID_50_ of PRRSV Lena. The experiment was performed at the IVI facilities and approved by the cantonal ethical committee for animal experiments (license #BE119/13). One animal was euthanized at 8 days post-infection (dpi) and the other two were kept for 52 dpi for long-term antiserum production. We took advantage of these sera for determining the time course of IFN-α production following PRRSV Lena infection.

### Stimulation of pDC

For pDC stimulation by PRRSV virions, 4 × 10^5^ enriched pDC/well of a 96-well plate were cultured in 200 μl and stimulated with various PRRSV strains at a multiplicity of infection (MOI) 0.1 TCID_50_/cell for 24 h at 39°C in 5% of CO_2_ atmosphere.

For pDC stimulation with infected cells, MØ and MARC-145 cells were first infected at an MOI of 0.1 TCID_50_/ml, or treated with mock cell extract as negative control. After virus adsorption for 1.5 h the cells were washed three times with warm PBS to remove the inoculum. Then, enriched pDC were added to the infected cells at 1 × 10^6^ cells/ml. Physical separation of infected MØ from pDC employed 24-well plates with trans-well inserts with 1 μm diameter pores (Corning, Sigma-Aldrich, Buchs, Switzerland and Becton Dickinson, Basel, Switzerland), into which 10^6^ enriched pDC were added in 100 μl medium.

As positive control for pDC stimulation, we used the TLR-9 ligand CpG oligodinucleotide D32 (10 μg/ml, Biosource, Int., Camarillo, CA, USA) or the TLR7 ligand Gardiquimod (10 μM, Invivogen, San Diego, CA, USA). IFN-α in supernatants was determined using ELISA as previously described (Guzylack-Piriou et al., 2004).

### Inhibition of pDC Sensing

In order to disrupt the cytoskeleton of freshly infected MØ, the cells were treated during 2 h with Nocodazole (10 μM; Sigma-Aldrich; interferes with microtubule polymerization) or Latrunculin B (3 μM; Sigma-Aldrich, Chemie GmbH, Buchs, Switzerland; inhibitor of actin polymerization), and then washed three times with warm medium before co-culture with enriched pDC. For the study of cell membrane functions GW4869 (Sigma-Aldrich) was used. This is a non-competitive neutral-sphingomyelinase (N-SMase) inhibitor preventing ceramide synthesis, which is widely used to block exosome generation (Kosaka et al., 2010). In order to test the effect of GW4869 on infected MØ, the cells were treated with culture medium containing 5, 10 or 20 μM GW4869. Appropriate dimethyl sulfoxide (DMSO, Sigma-Aldrich) controls ranging from 0.1 to 0.4% v/v were used. In order to evaluate integrin interaction between PRRSV-infected MØ and pDC, anti-CD11a mAb (clone MUC76A, Monoclonal Antibody Center, Washington State University, Pullman, WA, USA) was added to the co-culture at 10 or 30 μg/ml. In other experiments, anti-CD11a was used at 30 μg/ml in the medium of pDC in presence of exosome fractions from Lena-infected MØ.

### Flow Cytometry

For detection of the PRRSV N protein, MØ were harvested and fixed with 4% (w/v) paraformaldehyde (PFA) during 10 min at room temperature. The cells were then washed and permeabilized with 0.3% (w/v) Saponin in PBS in presence of SDOW17-A mAb (Rural Technologies, Inc., Brookings, SD, USA) for 15 min on ice, followed by incubation with an anti-mouse Alexa 488 fluochrome conjugate for 15 min (Life Technologies).

IFN-α-producing cells in PRRSV-infected MØ-pDC co-cultures were identified by intracellular IFN-α immunostaining after 12 h of culture. Brefeldin A (eBioscience, Austria) was added during the last 4 h. As control, enriched pDC were stimulated with CpG D32. The cells were stained for CD4 (mAb PT90A, VMRD Inc.) and CD172a (mAb 74-22-15a), fixed, and permeabilized in presence of anti-IFN-α mAb F17 (0.3 μg/ml; R&D Systems) as previously described (Baumann et al., 2013). The acquisition was performed on a FACSCanto (Becton Dickinson). For analysis, dead cells were excluded by electronic gating in forward/side scatter plots, followed by doublet discrimination. The pDC population was defined as CD172^low^CD4^high^, and MØ as CD172^high^CD4^neg^ (Summerfield and McCullough, 2009). Flowjo V.9.1 analysis software (Treestars, Inc., Ashland, OR, USA) was used.

### RNA Extraction and Reverse Transcription Quantitative Polymerase Chain Reaction

Total RNA from serum of Lena-infected pigs and PRRSV-infected MØ supernatant was extracted using the Nucleospin RNA II kit (Macherey-Nagel AG, Oensingen, Switzerland) following the manufacturer’s instructions, including DNase treatment and DNase inactivation steps. PRRSV reverse transcription quantitative polymerase chain reaction (RT-qPCR) was performed as previously described (Wernike et al., 2012). Briefly, RNA was added as template to the SuperScript III Platinium One-Step qRT-PCR System (Life Technologies) with ROX reference dye following the manufacturer’s instructions. The amplification was carried out using the 7500 Real-time PCR system (Applied Biosystem, Rotkreuz, Switzerland). The thermal cycling setup was 30 min at 50°C for the RT step, followed by the qPCR steps which included 2 min at 95°C of enzyme activation step, and 50 cycles of denaturation at 95°C for 15 s, annealing at 60°C for 30 s and extension at 72°C for 30 s. The quantification cycle (Cq) was determined and the results represented as the total number of cycles minus the Cq for each sample.

### Confocal Microscopy

MØ were differentiated in Lab-Tek II (Nunc; Milian, Geneva, Switzerland) at a density of 2.5 × 10^5^ cells/well in medium and infected as described above. 5 × 10^5^ enriched pDC were added and co-cultured with the MØ during 16 h at 39°C in 5% CO_2_ atmosphere. After incubation cells were washed with PBS and incubated with primary mAb against CD4 (clone PT90A; Monoclonal Antibody Center) for pDC labeling during 20 min at room temperature. After wash with cold PBS, the MØ cell membrane was stained with wheat germ agglutinin conjugated with Alexa Fluor633 (GWA-AF633; Life Technologies) during 1.5 min on ice, and washed twice with cold PBS. Then, the cells were fixed with 4% (w/v) PFA and permeabilized with 0.3% saponin (w/v) in PBS and PRRSV N protein was immunolabeled with SDOW17-A mAb during 20 min on ice. After a wash step with 0.1% (w/v) saponin in PBS, cells were incubated with secondary conjugated antibodies (Alexa488 or Alexa546; Life Technologies, Zug, Switzerland) in 0.3% saponin (w/v) in PBS for 20 min on ice. After washing with 0.1% (w/v) saponin in PBS, slides were mounted in Mowiol (Sigma-Aldrich). For confocal microscopy analysis a Leica TCS-SL confocal microscope and software (Leica Microsystem AG, Glattbrugg, Switzerland) were used. The image acquisitions were performed with the 63× oil-immersion objective; in order to give high-resolution images, the acquiring setting was performed with optimized voxel size and automatic threshold. The images were analyzed with Imaris 8.0.2 software (Bitplane AG, Zurich, Switzerland). To avoid false-positive emissions, different settings were applied including background subtraction, threshold applications, gamma correction, and maxima. The MØ and pDC surfaces were defined applying the surface module of the Imaris 8.0.2 software to GWA-AF633 and CD4-AF488 labeling, respectively. pDC tightly adhering to MØ were quantified after 16 h of co-culture. To this end, non-adherent cells were removed by washing the cultures twice with warm medium, and 20 different non-overlapping fields were counted in triplicate wells using confocal microscopy.

### Statistics

All experiments were done in triplicates and at least three times with cells from different blood donors. Figures and data collection analysis were done using GraphPad Prism 6 Software (GraphPad Software, Inc., San Diego, CA, USA). *P*-values were calculated by unpaired *t*-test, differences between groups were assessed by the Kruskas–Wallis analysis, and for individual differences the Mann–Whitney-*U*-test with Bonferroni correction as *post hoc* was employed. *P*-values lower than 0.05 were considered significant.

## Results

### PRRSV Strain Lena Induces IFN-α in Pigs But Not in pDC Cultures Stimulated with Virions

**FIGURE 1:**
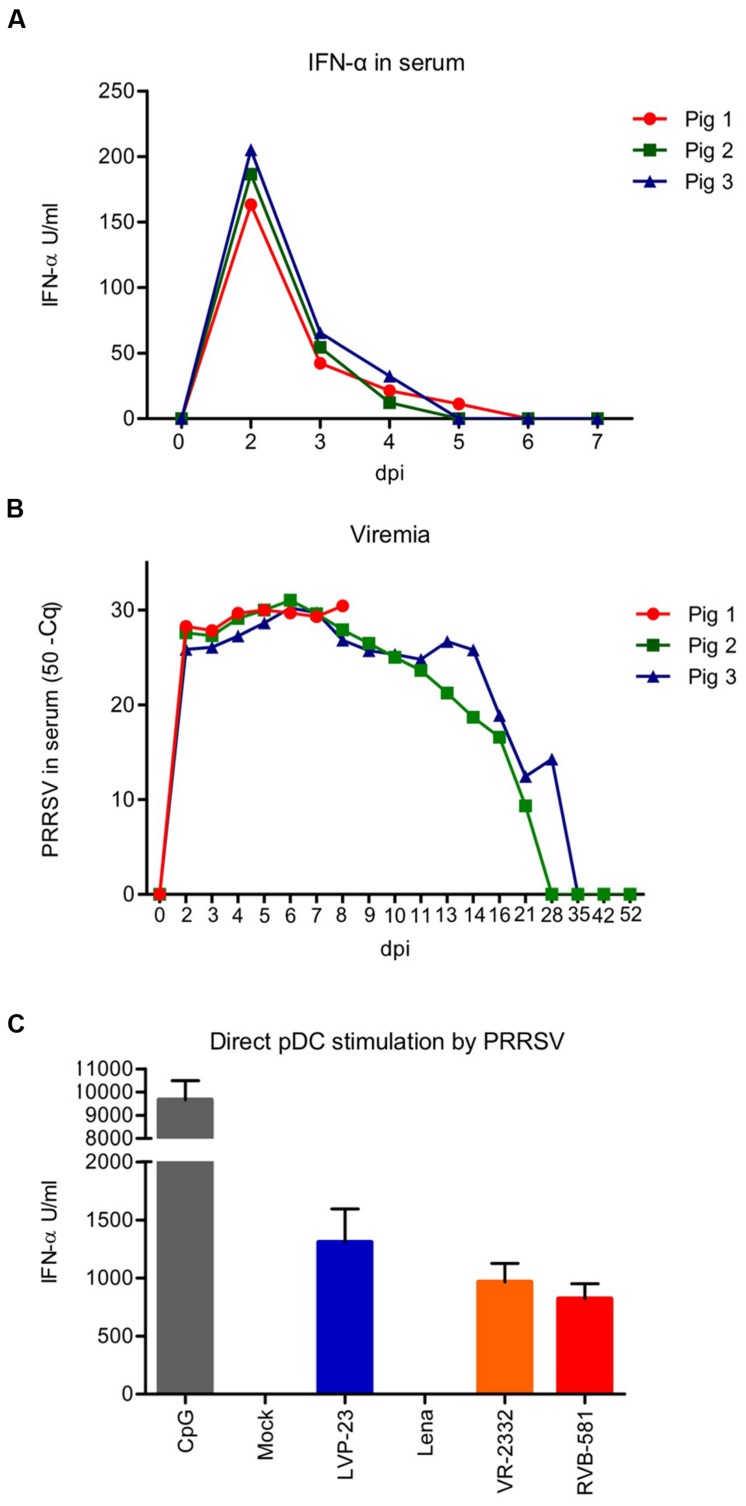
**The porcine reproductive and respiratory syndrome virus (PRRSV) Lena strain induces IFN-α in infected animals but cannot directly activate pDC *in vitro*.** **(A)** Serum IFN-α responses of pigs infected oronasally with PRRSV Lena (5 × 10^5^ TCID_50_ per animal), as determined by ELISA. **(B)** Viral RNA loads in the serum. The data was obtained using RT-qPCR for PRRSV ORF7 and is represented as the total number of cycles minus the quantification cycle (Cq)-value. **(C)** Comparative analysis of IFN-α production by enriched pDC in response to PRRSV-1 (LVP-23 and Lena) and PRRSV-2 strains (VR-2332 and RVB-581) after stimulation with a MOI of 0.1 TCID_50_/cell. CpG D32 was included as control. The supernatants were harvested after 20 h of incubation and IFN-α was quantified by ELISA. The data represent the mean IFN-α (U/ml) of triplicate cultures, with error bars representing the standard deviation. These results are representative of at least three independent experiments.

Considering that systemic IFN-α was observed following experimental infection with PRRSV (Liu et al., 2010), we analyzed sera collected during the first week of infection of pigs with the PRRSV Lena strain for IFN-α by ELISA (**Figure 1A**). In all animals a rapid and robust IFN-α production peaking at 2 dpi, with values ranging from 163 to 205 U/ml, was observed. Between 5 and 6 dpi the sera became negative, while viral RNA was detectable up to 28 dpi (**Figure 1B**). Both, type 1 and type 2 PRRSV isolates are able to induce IFN-α responses in pDC, as we have described previously (Baumann et al., 2013). The pDC may therefore represent an *in vivo* source for the systemic IFN-α response shown in **Figure 1A**. For this reason, we stimulated enriched pDC *in vitro* by PRRSV strains known to activated pDC including LVP-23, VR-2332, and RVB-581 as well as by the Lena strain. As shown in **Figure 1C**, all PRRSV strains promoted IFN-α secretion by pDC with the exception of Lena. Therefore, alternative pathways of IFN-α induction are required to explain the *in vivo* responses.

### PRRSV-Infected MØ Are Strong Activators of IFN-α Production by pDC in Co-culture

**FIGURE 2:**
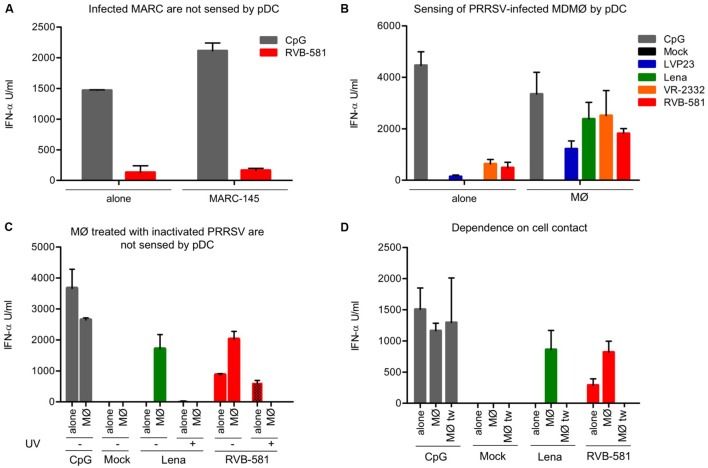
**Porcine reproductive and respiratory syndrome virus-infected MØ are strong inducers of IFN-α production by pDC.** **(A)** RVB-581-infected MARC-145 cells do not induce a stronger IFN-α secretion by pDC compared with the free viral particles effect on enriched pDC alone. MARC-145 cells were infected with RVB-581 for 90 min, washed and then co-cultured with enriched pDC. Stimulation of pDC by PRRSV virions (MOI 0.1 TCID_50_/cell) was used as control. **(B)** PRRSV-infected MØ are more potent activators of pDC as compared to virions (“alone”). Infection of cells was performed as in **(A)** using four different strains. **(C)** Stimulation of pDC by infected MØ but not by virions requires live virus. pDC were stimulated either with virions (“alone,” live or inactivated [UV]) or with infected MØ (“MØ”) as described in **(A)**. **(D)** pDC cultured in inserts of trans-well plates were either stimulated with virions (“alone”), by contact with infected MØ (“MØ”) or were physically separated from the infected MØ (“MØ tw”). CpG D32 was used as a control for strong pDC activation. All data are the result of experiments performed in triplicate with 20 h of stimulation, with error bars representing the standard deviation. The figures are representative of three **(A,B)** and two **(C,D)** different experiments.

With classical swine fever virus we have previously demonstrated that infected cells are much more potent at inducing IFN-α secretion by pDC when compared with direct stimulation by virions (Python et al., 2013). We therefore tested the ability of PRRSV-infected cells to activate pDC. RVB-581-infected MARC-145 co-cultured with pDC resulted in IFN-α responses, but there was no difference in level of IFN-α production between direct pDC stimulation with virions and pDC stimulation with PRRSV-infected MARC-145 cells (**Figure 2A**). This contrasted with PRRSV-infected MØ which stimulated at least 3.7 more IFN-α secretion when co-cultured with pDC as compared to direct stimulation by the same PRRSV strain (**Figure 2B**). Importantly, with this co-culture model the Lena strain induced high levels of IFN-α, although this strain was not able to activate pDC directly.

We have reported previously that UV-inactivated PRRSV is able to induce IFN-α secretion by direct stimulation of pDC (Baumann et al., 2013). Accordingly, UV-inactivated RVB-581 induced IFN-α using direct pDC stimulation. With the Lena strain, IFN-α induction was neither found in pDC infected with live nor with UV-inactivated virus (**Figure 2C**). In the co-culture setup, MØ treated with UV-inactivated virus did not induce IFN-α secretion in pDC, as opposed to MØ treated with live virus (**Figure 2C**). These results suggest that viral replication is required for pDC activation by infected MØ.

Based on previous work demonstrating that cell-to-cell contact is necessary for pDC sensing of infected cells (Python et al., 2013), we co-cultured pDC with PRRSV-infected MØ in trans-well culture dishes with 1 μm pore size. Our data show that PRRSV-infected MØ in direct contact with pDC promote a strong IFN-α secretion, while no response was found when pDC were physically separated from the MØ during the 24 h of co-culture (**Figure 2D**). It is important to note that pDC responses induced by a CpG control were similar if pDC where in the insert or in direct contact with MØ in the bottom of the well.

### During Co-Culture with PRRSV-Infected MØ Only pDC Produce IFN-α

**FIGURE 3:**
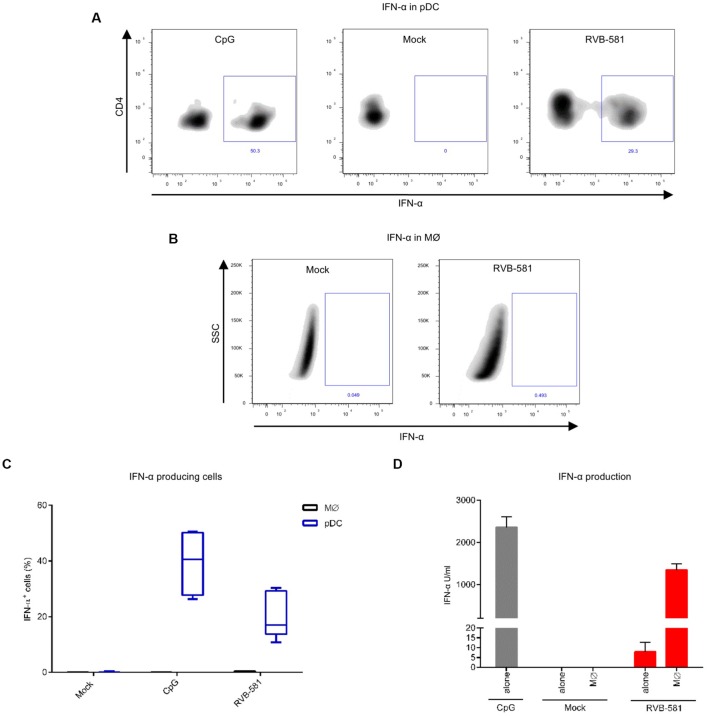
**IFN-α production is restricted to pDC during co-culture with PRRSV-infected MØ.** MØ were infected with PRRSV RVB-581 for 90 min, washed and then co-cultured with enriched pDC. Mock-treated MØ and CpG D32 stimulation were included as negative and positive controls, respectively. **(A)** Intracellular IFN-α staining of pDC (gated as CD172a^low^CD4^+^ cells) after CpG stimulation or co-culture with PRRSV-infected MØ. **(B)** Intracellular IFN-α staining of MØ (CD172a^+^CD4^-^ cells), harvested from the same co-culture as in **(A)**. **(C)** Boxplots made from nine replicates collected from three independent experiments showing the percentage of IFN-α expressing cells after stimulation as described in **(A,B)**. **(D)** IFN-α levels in supernatants collected from the experiments shown in **(A–C)**.

To determine the cellular source of IFN-α and identify the frequency of IFN-α producing cells, enriched pDC were co-cultured with PRRSV-infected MØ for 20 h and stained for CD4, CD172a and intracellular IFN-α. Dependent on the experiment, approximately 20–30% of pDC (CD172a^low^CD4^+^ cells) were positive for IFN-α staining, whereas MØ remained negative (**Figures 3A–C**). The frequency of IFN-α^+^ pDC was higher after stimulation with CpG relating to the higher levels of secreted IFN-α in such cultures, when compared to pDC stimulated with PRRSV-infected MØ (**Figures 3A,D**).

### Intact Cytoskeleton and Sphingomyelinase in MØ Are Required for PRRSV-Mediated pDC Stimulation

**FIGURE 4:**
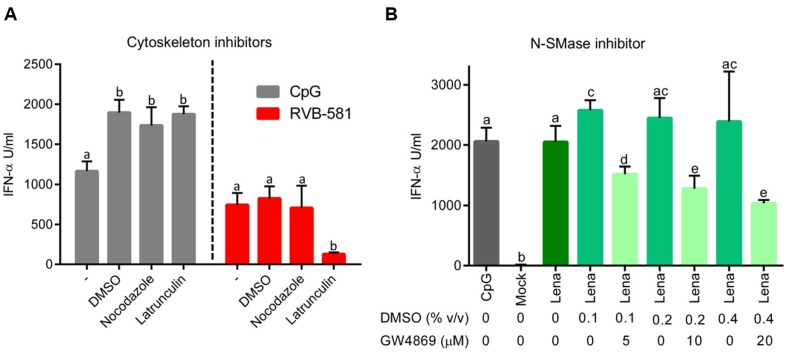
**Role of cytoskeleton and membrane ceramide in pDC activation by PRRSV-infected MØ.** **(A)** MØ were infected with PRRSV RVB-581 for 90 min, washed and then treated with the cytoskeleton inhibitors nocodazole (10 μM; interferes with microtubule polymerization) or latrunculin (3 μM; inhibitor of actin polymerization) or DMSO as control for 2 h at 39°C. After three washes, MØ were co-cultured with pDC. Control wells were stimulated with CpG D32. DMSO controls showed an increase in IFN-α secretion by pDC stimulated with CpG D32. **(B)** MØ were infected with PRRSV Lena for 90 min, washed and then co-cultured with pDC. Then DMSO controls or different concentrations of GW4869 (inhibitor N-SMase) at 5, 10, and 20 μM were added to the co-cultures. Mock-treated MØ and CpG D32 stimulation were included as controls. N-SMases are important in the metabolism of sphingomyelin and required to produce cell membrane ceramide and phosphocholine, which are important during intercellular interactions. For **(A,B)**, the IFN-α production was measured after 20 h of incubation in co-culture. The data represent mean values of three replicates with standard deviation, and represent three **(A)** and two **(B)** independent experiments. Different letters on top of the bars indicate significant difference based on the Mann–Whitney test (*P* < 0.05), whereas bars sharing same letters indicate no significant differences.

In order to investigate the pathways involved in communication between infected MØ and pDC, we first evaluated the role of the MØ cytoskeleton using inhibitors. To this end, MØ were infected with PRRSV for 90 min, washed and treated with nocodazole or latrunculin B for 2 h. After washing off the inhibitors, pDC were added in co-culture. Latrunculin B reduced pDC activation in terms of IFN-α production by fivefold, while no effect was found with nocodazole (**Figure 4A**). Importantly, in identical co-culture conditions in which the virus was replaced by CpG, latranculin B had no effect (**Figure 4A**). Furthermore, we demonstrated that the chemical compounds did not affect the viral replication. No major differences of PRRSV titres in supernatants from DMSO-treated (10^3.66^ TCID_50_/ml) and chemical-treated cells (10^3.5^ and 10^3.83^ TCID_50_/ml for nocodazole and latrunculin, respectively) were found.

Next we investigated a potential role of cell membrane ceramide which is required for exosome release. To this end, PRRSV-infected MØ were co-cultured with pDC in presence of GW4869, a N-SMase inhibitor preventing ceramide synthesis. We observed a GW4869-dose dependent effect on pDC activation by PRRSV-infected MØ, with up to 50% reduction of IFN-α responses at 20 μM (**Figure 4B**). As GW4869 was present during the whole co-culture incubation, further investigations were carried out to evaluate negative effects on pDC functions and viral replication in MØ. To this end, pDC were stimulated with the TLR9 or TLR7 ligands CpG or Gardiquimod, respectively, in presence of GW4869 at different concentrations. The pDC functions were not significantly impaired by the N-SMase inhibitor (**Supplementary Figures S1A,B**). Also no effect on PRRSV infection and replication in MØ were detected. GW4869 did not affect PRRSV infection of MØ, when present only during virus adsorption (**Supplementary Figure S1C**) or after adsorption (**Supplementary Figure S1D**). Furthermore, the drug affected neither the virus titer nor viral RNA in supernatants of PRRSV-infected MØ (**Supplementary Figures S1E,F**).

### Evidence for a Role of Exosomes in pDC Stimulation by PRRSV-Infected MØ

**FIGURE 5:**
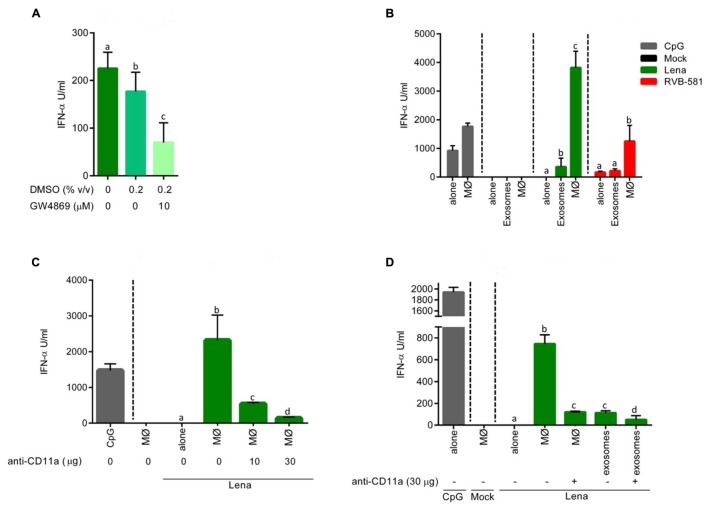
**pDC stimulation by PRRSV-infected MØ is dependent on integrin-mediated cell contact.** **(A)** Stimulation of pDC by MØ-derived exosomes. MØ were infected with PRRSV Lena for 90 min followed by wash steps and addition of fresh medium. After 18 h of culture, exosome fractions were isolated and used to stimulate enriched pDC during 20 h at 39°C. The N-SMase inhibitor GW4869 was added at 10 μM, DMSO alone was added at 0.2% (v/v) or the cells were left untreated. **(B)** Stimulation of pDC by infected MØ is superior to exosome stimulation. pDC were stimulated with PRRSV (Lena or RVB-581) virions at MOI 0.1 TCID_50_/ml (“alone”), with PRRSV-infected (Lena or RVB-581) MØ, with exosome fractions isolated from supernatant of PRRSV-infected (Lena or RVB-581) MØ; CpG D32 was included as control of pDC activation. **(C)** pDC stimulation by infected MØ involves ITGAL (CD11a). pDC were stimulated with mock-treated MØ, with Lena virions at MOI 0.1 TCID_50_/ml (“alone”) and with Lena-infected MØ. Anti-CD11a mAb was added to the co-cultures at 10 or 30 μg/ml as indicated. CpG D32 was included as control for pDC activation. **(D)** Effect of anti-CD11a on pDC stimulation by infected MØ or exosomes. pDC were stimulated with mock-treated MØ, with Lena virions at MOI 0.1 TCID_50_/ml (“alone”), with Lena-infected MØ or with the exosome fraction isolated from Lena-infected MØ. Anti-CD11a mAb was added to the co-culture and to the exosome fraction stimulation at 30 μg/ml. CpG D32 was included as control for pDC activation. Experiments were done in triplicates, and represent three **(A,B)** or two **(C,D)** different experiments. Different letters on top of the bars indicate significant difference based on the Mann–Whitney test (*P* < 0.05), whereas bars sharing same letters indicate no significant differences.

As GW4869 has been described as inhibitor of exosome release (Kosaka et al., 2010), we investigated if exosome fractions from the supernatant of PRRSV-infected MØ can stimulate pDC and if this is inhibited by GW4869. As shown in **Figure 5A**, such exosomes preparations induce IFN-α in pDC, which is inhibited when the MØ are treated with GW4869. Since the exosome preparations also contained live PRRSV, we compared the levels of pDC activation by exosome preparations of RVB-581- and Lena-infected MØ with virion-induced pDC activation by the same viruses (**Figure 5B**). The results show that the exosome fraction of Lena-infected cells but not Lena virions themselves are able to activate pDC.

### Integrins Are Involved in Sensing of Infected MØ by pDC

The above results of **Figure 5B** demonstrate that co-culture of pDC with infected MØ is much more efficient than stimulation by exosomes in terms of activating pDC. This is also supported by the observation that no IFN-α is induced when MØ are physically separated using trans-well culture dishes (**Figure 2D**). We therefore hypothesized that tight contact between the two cell types is required. In order to elaborate on this, we tested the effect of antibodies against the integrin α chain (ITGAL, LFA-1, or CD11a), which plays a central role in leukocyte intercellular adhesion. In fact, when PRRSV-infected MØ were co-cultured with pDC in presence of anti-CD11a mAb a dose-dependent impairment of IFN-α release from pDC was observed (**Figure 5C**). ITGAL blocking not only potently impaired pDC stimulation by PRRSV-infected MØ, but also had a statistically significant effect on stimulation of pDC by exosomes, although the levels of IFN-α were much lower (**Figure 5D**). These results indicate that ITGAL-mediated intercellular adhesion is required for efficient sensing of PRRSV-infected MØ.

### pDC Are in Intimate Contact with PRRSV-Infected MØ during the Co-culture

**FIGURE 6:**
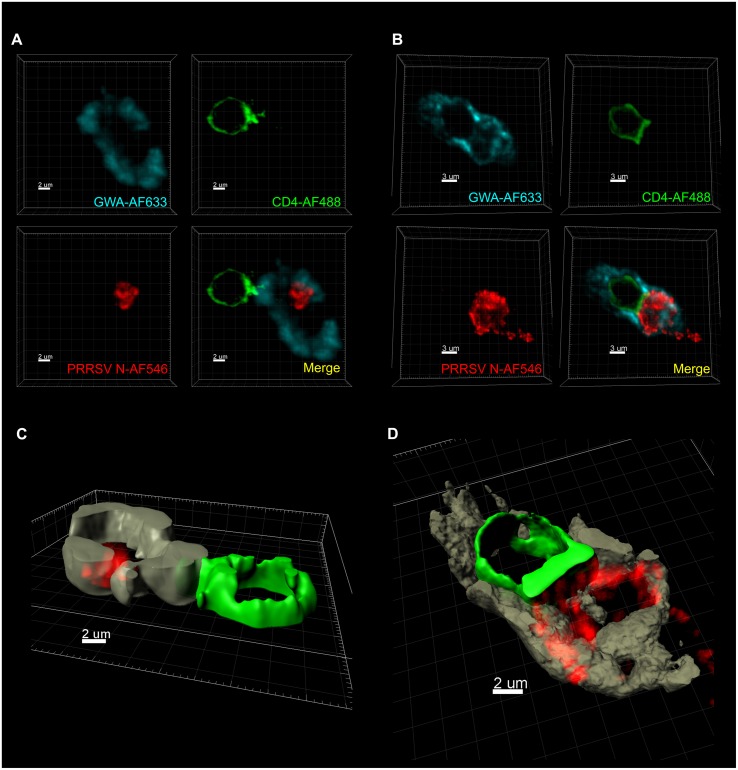
**Porcine reproductive and respiratory syndrome virus-infected MØ are in tight contact with pDC during co-culture.** MØ were infected with PRRSV Lena for 90 min followed by wash steps and then co-cultured with pDC during another 16 h. Then MØ cell membrane was labeled with GWA-AF633 (turquoise in **A**,**B**; brown in **C,D**), pDC surfaces were labeled with CD4-AF488 (green), and PRRSV N protein (red) was stained with SDOW17-A-AF546. 3D scans were acquired using confocal microscopy. **(A,B)** Two different types of the cell-to-cell contact were observed. All micrographs are 3D Blend images with threshold subtraction and gamma correction set as in mock-treated MØ. The images were acquired with zoom together with optimum resolution format to provide the best voxel size for each employed fluorochrome. **(C**,**D)** 3D images were created using the surface module of the Imaris software applied on the labeling of GWA-AF633 and CD4-AF488 for the MØ and the pDC surfaces, respectively.

In order to visualize this intercellular interaction, PRRSV-infected MØ were co-cultured with pDC, and after 16 h of incubation, cell membranes of MØ and pDC as well as PRRSV N protein were fluorochrome labeled. These experiments showed that pDC and infected MØ get in close and tight contact during the co-culture. We observed a polarization of CD4 at the area of contact between the two cell membranes (**Figure 6A**). In cells with tight contact, we also found that PRRSV N protein appears to be directed toward pDC (**Figure 6B**). The Imaris software Surface Module served to create 3D images confirming the above descriptions and demonstrating a MØ “embracing” a pDC (**Figures 6C,D**). It is important to note that no PRRSV N protein was detected inside pDC. The frequency of MØ in tight contact with pDC, such as shown in **Figure 6A**, was found to be 1.3% of all counted MØ in a given field, when analyzed at 16 h after initiation of the co-cultures. The maximum theoretical value for such interactions was calculated to be around 10% as the ratio of pDC to MØ was 1:10 in the co-cultures. Such interactions were also found in pDC and MØ in uninfected cultures at a similar frequency.

## Discussion

Porcine reproductive and respiratory syndrome virus is a very weak inducer of IFN-I responses following infection of PAM, MoDC or MØ *in vitro* (Lee et al., 2004; Zhang et al., 2012; Garcia-Nicolas et al., 2014). In pDC, however, the two PRRSV genotypes are able to induce robust IFN-α responses by virion-mediated stimulation of the TLR7 pathway (Baumann et al., 2013). In the present study, we found that the PRRSV-1 subtype 3 strain Lena is an exception in not being able to activate pDC directly. We currently have no explanation for this biological difference of Lena to other PRRSV strains. Considering that PRRSV does not need to replicate to activate pDC, the lack of stimulation is unlikely to be caused by inhibitory non-structural proteins of the virus. Future studies should address a possible role for the envelope glycoproteins of Lena, as this strain has been shown to differ in tropism when compared to other PRRSV-1 strains (Frydas et al., 2013). It is possible that these differences result in a reduced ability of pDC to endocytose the virus for stimulation of TLR7 or alternatively that surface glycoproteins of the Lena strain have inhibitory activity on pDC.

Despite this lack of pDC activation, similar to other PRRSV strains, a short but high-level systemic IFN-α response during the first week of infection was observed in pigs infected with the Lena strain. Our *in vitro* studies, demonstrating that PRRSV-infected MØ are far more efficient at stimulating pDC than virions, resulting in almost maximum IFN-α found with this cell type, represent a possible explanation for this apparent discrepancy.

These findings relate to previous reports with other viruses including human immunodeficiency virus, hepatitis C virus (HCV), Venezuelan equine encephalitis virus, classical swine fever virus, foot-and-mouth disease virus and Dengue virus, demonstrating that virus-infected cells are often more potent activators of pDC responses, as compared to free viral particles (Megjugorac et al., 2007; Takahashi et al., 2010; Python et al., 2013; Zhang et al., 2013; Decembre et al., 2014; Frenz et al., 2014). This increasing number of reports and the sticking efficiency of this process support an *in vivo* relevance for this mode of antiviral innate response.

As described for other viruses this pathway of pDC activation is dependent on cell-to-cell contact, in which the integrity of actin microfilaments in the cytoskeleton of the MØ are important. In addition, active N-SMase was required in the infected cells, indicating a role for membrane ceramide. In order to inhibit N-SMase, we employed GW4869 which was used as exosome release inhibitor (Kosaka et al., 2010), pointing on a possible role for such microvesicles, as has been described for HCV and Lymphocytic Choriomeningitis Virus (Dreux et al., 2012; Wieland et al., 2014). Such exosomes have been postulated to transfer viral RNA to the TLR7 compartment of pDC (Dreux et al., 2012; Wieland et al., 2014). Nevertheless, while we were able to demonstrate that in contrast to Lena virions, the exosome-rich fraction of Lena-infected MØ is able to stimulate pDC, our results also show that pDC activation by PRRSV-infected cells is much stronger than activation by the exosome fraction. In addition, GW4869 only partially inhibited pDC activation by PRRSV-infected cells, questioning whether exosomes containing viral RNA are the main inducers of pDC responses in our model. In support of this, blocking the integrin ITGAL (CD11a) abrogated the ability of pDC to sense PRRSV-infected cells, while it had no effect on pDC activation mediated by the exosome-fraction. ITGAL plays a central role in leucocyte intercellular interactions through binding to intercellular adhesion molecules 1-3 (ICAM; Hibbs et al., 1991; Mukai et al., 1999; Whitcup et al., 1999; Hagberg et al., 2011). This interaction has various functions in the immune response, one of them being the interaction between T cells and antigen-presenting cells, which also requires intimate intercellular contact for the formation of the “immunological synapse” (Hosseini et al., 2009; Manikwar et al., 2012). It has also been described that NK cells promote IFN-I responses through direct contact with pDC, with ITGAL (LFA-1) being involved (Hagberg et al., 2011). During our confocal microscopy analysis, we found numerous strikingly tight interactions between infected MØ and pDC. We speculate that such interactions could be involved during the transfer of viral RNA to the TLR7 compartment of pDC. In lymphoid tissues from PRRSV-infected pigs, cells expressing IFN-α in close proximity to PRRSV-infected cells have been described (Barranco et al., 2012), which support this idea. Clearly, future studies are required to investigate the role and the biology of the interaction of pDC with infected cells.

## Conclusion

While PRRSV clearly suppresses IFN-I induction in its target cells used for replication, our data suggest that during the interaction with pDC the host can make considerable quantities of IFN-α. This and the robust systemic IFN-α response are questioning the concept of PRRSV as a virus, which is not well sensed by the innate immune system (Albina et al., 1998; Labarque et al., 2000; Darwich et al., 2010). Having said that, the immunology of PRRSV is quite complex and the virus clearly has established many mechanisms to prevent efficient immune responses resulting in a slow resolution of the infection (Xiao et al., 2004; Chen et al., 2010; Patel et al., 2010). More generally, the ability of pDC to sense efficiently virus-infected cells and not only free virions offers the innate immune system a possibility to react to viruses before they are released from the infected cells. In addition to the antiviral activity, this early boost of the IFN system will alert the immune system and initiate the induction of adaptive immune responses. On the other hand, IFN type I responses also represent a double-edge sword as they can be associated with severe immunopathology such as observed during classical swine fever virus infection (Summerfield and Ruggli, 2015) or many other viral diseases (Tomasello et al., 2014). Deleterious effects are particularly observed with prolonged systemic IFN type I responses found in certain chronic virus infections (Summerfield, 2012; Tomasello et al., 2014). Of particular relevance for PRRS, could be a possible association between IFN type I responses in the lung and secondary bacterial infections as reported for other viruses (Tomasello et al., 2014). Clearly, future *in vivo* studies are required to address the role of pDC and IFN-α in the immune response against PRRSV.

## Author Contributions

OG-N: study design, execution of the experiments, data analysis, writing of the manuscript; GA: study design (partly), execution of the experiments, data analysis; JR: study design (partly), execution of the experiments; CS: study design (partly), execution of the experiments, data analysis; KM: study design, execution of the experiments, data analysis; NR: study design, writing of the manuscript; AS: overall concept and study design, writing of the manuscript.

## Conflict of Interest Statement

The authors declare that the research was conducted in the absence of any commercial or financial relationships that could be construed as a potential conflict of interest.

## Supplementary Material

**FIGURE S1 | GW4869, a non-competitive inhibitor of N-SMase neither affects TLR9 nor TLR7 signaling in pDC nor PRRSV infection and replication in MØ.** pDC were stimulated with TLR9 ligand CpG D32 **(A)** or with TLR7 ligand Gardiquimod **(B)** and treated with DMSO control at different concentrations (0.1, 0.2, and 0.4% v/v), in presence or absence of GW4869 at 5, 10, and 20 μM. These results demonstrate that IFN-α production by pDC was not affected by N-SMase inhibitor (GW4869) at any tested concentration. **(C)** MØ were infected with PRRSV Lena in the presence of GW4869 during the virus adsorption incubation time. After 90 min, the cells were washed and incubated for 16 h before PRRSV N expressing cells were quantified by flow cytometry; demonstrating that this inhibitor does not affect the virus entry into the target cells. In **(D)** GW4869 was present only after the 90 min adsorption period, which shows that GW4869 has no effect on the viral replication in MØ. **(E)** PRRSV virus titres in supernatant collected from **(C)**. **(F)** PRRSV RNA levels in supernatants of **(C)** quantified using RT-qPCR. Both **(E,F)** confirm observations previously done in **(D)**. Significant differences between conditions are indicated by asterisks based on the Mann–Whitney test (^∗^*P* < 0.05).

## References

[B1] Albina E. Carrat C. Charley B. (1998). Interferon-alpha response to swine arterivirus (PoAV), the porcine reproductive and respiratory syndrome virus. *J. Interferon Cytokine Res.* 18 485–490. 10.1089/jir.1998.18.485 9712364

[B2] Barranco I. Gomez-Laguna J. Rodriguez-Gomez I. M. Quereda J. J. Salguero F. J. Pallares F. J. (2012). Immunohistochemical expression of IL-12, IL-10, IFN-alpha and IFN-gamma in lymphoid organs of porcine reproductive and respiratory syndrome virus-infected pigs. *Vet. Immunol. Immunopathol.* 149 262–271. 10.1016/j.vetimm.2012.07.011 22889555

[B3] Baumann A. Mateu E. Murtaugh M. P. Summerfield A. (2013). Impact of genotype 1 and 2 of porcine reproductive and respiratory syndrome viruses on interferon-alpha responses by plasmacytoid dendritic cells. *Vet. Res.* 44:33 10.1186/1297-9716-44-33 PMC3672080 23675981

[B4] Buddaert W. Van Reeth K. Pensaert M. (1998). In vivo and in vitro interferon (IFN) studies with the porcine reproductive and respiratory syndrome virus (PRRSV). *Adv. Exp. Med. Biol.* 440 461–467. 10.1007/978-1-4615-5331-1_59 9782316

[B5] Carrasco C. P. Rigden R. C. Schaffner R. Gerber H. Neuhaus V. Inumaru S. (2001). Porcine dendritic cells generated in vitro: morphological, phenotypic and functional properties. *Immunology* 104 175–184. 10.1046/j.1365-2567.2001.01299.x 11683958 PMC1783296

[B6] Chen Z. Lawson S. Sun Z. Zhou X. Guan X. Christopher-Hennings J. (2010). Identification of two auto-cleavage products of nonstructural protein 1 (nsp1) in porcine reproductive and respiratory syndrome virus infected cells: nsp1 function as interferon antagonist. *Virology* 398 87–97. 10.1016/j.virol.2009.11.033 20006994 PMC7111964

[B7] Collins J. E. Benfield D. A. Christianson W. T. Harris L. Hennings J. C. Shaw D. P. (1992). Isolation of swine infertility and respiratory syndrome virus (isolate ATCC VR-2332) in North America and experimental reproduction of the disease in gnotobiotic pigs. *J. Vet. Diagn. Invest.* 4 117–126. 10.1177/104063879200400201 1616975

[B8] Darwich L. Diaz I. Mateu E. (2010). Certainties, doubts and hypotheses in porcine reproductive and respiratory syndrome virus immunobiology. *Virus Res.* 154 123–132. 10.1016/j.virusres.2010.07.017 20659507

[B9] Decembre E. Assil S. Hillaire M. L. Dejnirattisai W. Mongkolsapaya J. Screaton G. R. (2014). Sensing of immature particles produced by dengue virus infected cells induces an antiviral response by plasmacytoid dendritic cells. *PLoS Pathog.* 10:e1004434 10.1371/journal.ppat.1004434 PMC4207819 25340500

[B10] Done S. H. Paton D. J. (1995). Porcine reproductive and respiratory syndrome: clinical disease, pathology and immunosuppression. *Vet. Rec.* 136 32–35. 10.1136/vr.136.2.32 7709569

[B11] Dreux M. Garaigorta U. Boyd B. Decembre E. Chung J. Whitten-Bauer C. (2012). Short-range exosomal transfer of viral RNA from infected cells to plasmacytoid dendritic cells triggers innate immunity. *Cell Host Microbe* 12 558–570. 10.1016/j.chom.2012.08.010 23084922 PMC3479672

[B12] Frenz T. Graalmann L. Detje C. N. Doring M. Grabski E. Scheu S. (2014). Independent of plasmacytoid dendritic cell (pDC) infection, pDC triggered by virus-infected cells mount enhanced type I IFN responses of different composition as opposed to pDC stimulated with free virus. *J. Immunol.* 193 2496–2503. 10.4049/jimmunol.1400215 25070849

[B13] Frydas I. S. Verbeeck M. Cao J. Nauwynck H. J. (2013). Replication characteristics of porcine reproductive and respiratory syndrome virus (PRRSV) European subtype 1 (Lelystad) and subtype 3 (Lena) strains in nasal mucosa and cells of the monocytic lineage: indications for the use of new receptors of PRRSV (Lena). *Vet. Res.* 44 73 10.1186/1297-9716-44-73 PMC3849772 24007551

[B14] Garcia-Nicolas O. Baumann A. Vielle N. J. Gomez-Laguna J. Quereda J. J. Pallares F. J. (2014). Virulence and genotype-associated infectivity of interferon-treated macrophages by porcine reproductive and respiratory syndrome viruses. *Virus Res.* 179 204–211. 10.1016/j.virusres.2013.08.009 24220223

[B15] Guo B. Lager K. M. Henningson J. N. Miller L. C. Schlink S. N. Kappes M. A. (2013). Experimental infection of United States swine with a Chinese highly pathogenic strain of porcine reproductive and respiratory syndrome virus. *Virology* 435 372–384. 10.1016/j.virol.2012.09.013 23079105 PMC7111980

[B16] Guzylack-Piriou L. Balmelli C. McCullough K. C. Summerfield A. (2004). Type-A CpG oligonucleotides activate exclusively porcine natural interferon-producing cells to secrete interferon-alpha, tumour necrosis factor-alpha and interleukin-12. *Immunology* 112 28–37. 10.1111/j.1365-2567.2004.01856.x 15096181 PMC1782461

[B17] Guzylack-Piriou L. Bergamin F. Gerber M. McCullough K. C. Summerfield A. (2006). Plasmacytoid dendritic cell activation by foot-and-mouth disease virus requires immune complexes. *Eur. J. Immunol.* 36 1674–1683. 10.1002/eji.200635866 16783856

[B18] Hagberg N. Berggren O. Leonard D. Weber G. Bryceson Y. T. Alm G. V. (2011). IFN-alpha production by plasmacytoid dendritic cells stimulated with RNA-containing immune complexes is promoted by NK cells via MIP-1beta and LFA-1. *J. Immunol.* 186 5085–5094. 10.4049/jimmunol.1003349 21430220

[B19] Hibbs M. L. Xu H. Stacker S. A. Springer T. A. (1991). Regulation of adhesion of ICAM-1 by the cytoplasmic domain of LFA-1 integrin beta subunit. *Science* 251 1611–1613. 10.1126/science.1672776 1672776

[B20] Holtkamp D. J. Kliebenstein J. B. Neumann E. J. Zimmerman J. J. Rotto H. F. Yoder T. K. (2013). Assessment of the economic impact of porcine reproductive and respiratory syndrome virus on United States pork producers. *J. Swine Health Product.* 21 72–84.

[B21] Hosseini B. H. Louban I. Djandji D. Wabnitz G. H. Deeg J. Bulbuc N. (2009). Immune synapse formation determines interaction forces between T cells and antigen-presenting cells measured by atomic force microscopy. *Proc. Natl. Acad. Sci. U.S.A.* 106 17852–17857. 10.1073/pnas.0905384106 19822763 PMC2764924

[B22] Karniychuk U. U. Geldhof M. Vanhee M. Van Doorsselaere J. Saveleva T. A. Nauwynck H. J. (2010). Pathogenesis and antigenic characterization of a new East European subtype 3 porcine reproductive and respiratory syndrome virus isolate. *BMC Vet. Res.* 6:30 10.1186/1746-6148-6-30 PMC2898778 20525333

[B23] Kosaka N. Iguchi H. Yoshioka Y. Takeshita F. Matsuki Y. Ochiya T. (2010). Secretory mechanisms and intercellular transfer of microRNAs in living cells. *J. Biol. Chem.* 285 17442–17452. 10.1074/jbc.M110.107821 20353945 PMC2878508

[B24] Labarque G. G. Nauwynck H. J. Van Reeth K. Pensaert M. B. (2000). Effect of cellular changes and onset of humoral immunity on the replication of porcine reproductive and respiratory syndrome virus in the lungs of pigs. *J. Gen. Virol.* 81(Pt 5), 1327–1334. 10.1099/0022-1317-81-5-1327 10769076

[B25] Lee S. M. Schommer S. K. Kleiboeker S. B. (2004). Porcine reproductive and respiratory syndrome virus field isolates differ in in vitro interferon phenotypes. *Vet. Immunol. Immunopathol.* 102 217–231. 10.1016/j.vetimm.2004.09.009 15507307 PMC7112598

[B26] Liu Y. Shi W. Zhou E. Wang S. Hu S. Cai X. (2010). Dynamic changes in inflammatory cytokines in pigs infected with highly pathogenic porcine reproductive and respiratory syndrome virus. *Clin. Vaccine Immunol.* 17 1439–1445. 10.1128/CVI.00517-09 20631336 PMC2944458

[B27] Liu Y. J. (2005). IPC: professional type 1 interferon-producing cells and plasmacytoid dendritic cell precursors. *Annu. Rev. Immunol.* 23 275–306. 10.1146/annurev.immunol.23.021704.115633 15771572

[B28] Lopez O. J. Osorio F. A. (2004). Role of neutralizing antibodies in PRRSV protective immunity. *Vet. Immunol. Immunopathol.* 102 155–163. 10.1016/j.vetimm.2004.09.005 15507302

[B29] Manikwar P. Kiptoo P. Badawi A. H. Buyuktimkin B. Siahaan T. J. (2012). Antigen-specific blocking of CD4-specific immunological synapse formation using BPI and current therapies for autoimmune diseases. *Med. Res. Rev.* 32 727–764. 10.1002/med.20243 21433035 PMC4441537

[B30] Megjugorac N. J. Jacobs E. S. Izaguirre A. G. George T. C. Gupta G. Fitzgerald-Bocarsly P. (2007). Image-based study of interferongenic interactions between plasmacytoid dendritic cells and HSV-infected monocyte-derived dendritic cells. *Immunol. Invest.* 36 739–761. 10.1080/08820130701715845 18161527

[B31] Meier W. A. Galeota J. Osorio F. A. Husmann R. J. Schnitzlein W. M. Zuckermann F. A. (2003). Gradual development of the interferon-gamma response of swine to porcine reproductive and respiratory syndrome virus infection or vaccination. *Virology* 309 18–31. 10.1016/S0042-6822(03)00009-6 12726723

[B32] Mukai S. Kagamu H. Shu S. Plautz G. E. (1999). Critical role of CD11a (LFA-1) in therapeutic efficacy of systemically transferred antitumor effector T cells. *Cell Immunol.* 192 122–132. 10.1006/cimm.1998.1439 10087180

[B33] Nelsen C. J. Murtaugh M. P. Faaberg K. S. (1999). Porcine reproductive and respiratory syndrome virus comparison: divergent evolution on two continents. *J. Virol.* 73 270–280. 9847330 10.1128/jvi.73.1.270-280.1999 PMC103831

[B34] Patel D. Nan Y. Shen M. Ritthipichai K. Zhu X. Zhang Y. J. (2010). Porcine reproductive and respiratory syndrome virus inhibits type I interferon signaling by blocking STAT1/STAT2 nuclear translocation. *J. Virol.* 84 11045–11055. 10.1128/JVI.00655-10 20739522 PMC2953160

[B35] Python S. Gerber M. Suter R. Ruggli N. Summerfield A. (2013). Efficient sensing of infected cells in absence of virus particles by plasmacytoid dendritic cells is blocked by the viral ribonuclease E(rns.). *PLoS Pathog.* 9:e1003412 10.1371/journal.ppat.1003412 PMC3681750 23785283

[B36] Shi M. Lam T. T. Hon C. C. Hui R. K. Faaberg K. S. Wennblom T. (2010). Molecular epidemiology of PRRSV: a phylogenetic perspective. *Virus Res.* 154 7–17. 10.1016/j.virusres.2010.08.014 20837072

[B37] Solano G. I. Segales J. Collins J. E. Molitor T. W. Pijoan C. (1997). Porcine reproductive and respiratory syndrome virus (PRRSv) interaction with *Haemophilus parasuis*. *Vet. Microbiol.* 55 247–257. 10.1016/S0378-1135(96)01325-9 9220620 PMC7117440

[B38] Stadejek T. Oleksiewicz M. B. Scherbakov A. V. Timina A. M. Krabbe J. S. Chabros K. (2008). Definition of subtypes in the European genotype of porcine reproductive and respiratory syndrome virus: nucleocapsid characteristics and geographical distribution in Europe. *Arch. Virol.* 153 1479–1488. 10.1007/s00705-008-0146-2 18592131

[B39] Stadejek T. Stankevicius A. Murtaugh M. P. Oleksiewicz M. B. (2013). Molecular evolution of PRRSV in Europe: current state of play. *Vet. Microbiol.* 165 21–28. 10.1016/j.vetmic.2013.02.029 23528651

[B40] Summerfield A. (2012). Viewpoint: factors involved in type I interferon responses during porcine virus infections. *Vet. Immunol. Immunopathol.* 148 168–171. 10.1016/j.vetimm.2011.03.011 21458079

[B41] Summerfield A. McCullough K. C. (2009). The porcine dendritic cell family. *Dev. Comp. Immunol.* 33 299–309. 10.1016/j.dci.2008.05.005 18582937 PMC7103208

[B42] Summerfield A. Ruggli N. (2015). Immune responses against classical swine fever virus: between ignorance and lunacy. *Front. Vet. Sci.* 2:10 10.3389/fvets.2015.00010 PMC4672165 26664939

[B43] Takahashi K. Asabe S. Wieland S. Garaigorta U. Gastaminza P. Isogawa M. (2010). Plasmacytoid dendritic cells sense hepatitis C virus-infected cells, produce interferon, and inhibit infection. *Proc. Natl. Acad. Sci. U.S.A.* 107 7431–7436. 10.1073/pnas.1002301107 20231459 PMC2867703

[B44] Thanawongnuwech R. Thacker B. Halbur P. Thacker E. L. (2004). Increased production of proinflammatory cytokines following infection with porcine reproductive and respiratory syndrome virus and *Mycoplasma hyopneumoniae*. *Clin. Diagn. Lab. Immunol.* 11 901–908. 10.1128/CDLI.11.5.901-908.2004 15358650 PMC515260

[B45] Tomasello E. Pollet E. Vu Manh T. P. Uze G. Dalod M. (2014). Harnessing mechanistic knowledge on beneficial versus deleterious IFN-I effects to design innovative immunotherapies targeting cytokine activity to specific cell types. *Front. Immunol.* 5:526 10.3389/fimmu.2014.00526 PMC4214202 25400632

[B46] Van Breedam W. Delputte P. L. Van Gorp H. Misinzo G. Vanderheijden N. Duan X. (2010). Porcine reproductive and respiratory syndrome virus entry into the porcine macrophage. *J. Gen. Virol.* 91(Pt 7), 1659–1667. 10.1099/vir.0.020503-0 20410315

[B47] Van Reeth K. Van Gucht S. Pensaert M. (2002). In vivo studies on cytokine involvement during acute viral respiratory disease of swine: troublesome but rewarding. *Vet. Immunol. Immunopathol.* 87 161–168. 10.1016/S0165-2427(02)00047-8 12072230 PMC7119797

[B48] Wernike K. Hoffmann B. Dauber M. Lange E. Schirrmeier H. Beer M. (2012). Detection and typing of highly pathogenic porcine reproductive and respiratory syndrome virus by multiplex real-time rt-PCR. *PLoS ONE* 7:e38251 10.1371/journal.pone.0038251 PMC3387184 22768042

[B49] Whitcup S. M. Chan C. C. Kozhich A. T. Magone M. T. (1999). Blocking ICAM-1 (CD54) and LFA-1 (CD11a) inhibits experimental allergic conjunctivitis. *Clin. Immunol.* 93 107–113. 10.1006/clim.1999.4775 10527686

[B50] Wieland S. F. Takahashi K. Boyd B. Whitten-Bauer C. Ngo N. de la Torre J. C. (2014). Human plasmacytoid dendritic cells sense lymphocytic choriomeningitis virus-infected cells in vitro. *J. Virol.* 88 752–757. 10.1128/JVI.01714-13 24155390 PMC3911694

[B51] Xiao Z. Batista L. Dee S. Halbur P. Murtaugh M. P. (2004). The level of virus-specific T-cell and macrophage recruitment in porcine reproductive and respiratory syndrome virus infection in pigs is independent of virus load. *J. Virol.* 78 5923–5933. 10.1128/JVI.78.11.5923-5933.2004 15140990 PMC415812

[B52] Yoo D. Song C. Sun Y. Du Y. Kim O. Liu H. C. (2010). Modulation of host cell responses and evasion strategies for porcine reproductive and respiratory syndrome virus. *Virus Res.* 154 48–60. 10.1016/j.virusres.2010.07.019 20655963 PMC7114477

[B53] Zhang H. Guo X. Nelson E. Christopher-Hennings J. Wang X. (2012). Porcine reproductive and respiratory syndrome virus activates the transcription of interferon alpha/beta (IFN-alpha/beta) in monocyte-derived dendritic cells (Mo-DC). *Vet. Microbiol.* 159 494–498. 10.1016/j.vetmic.2012.04.025 22592217 PMC7127654

[B54] Zhang S. Kodys K. Babcock G. J. Szabo G. (2013). CD81/CD9 tetraspanins aid plasmacytoid dendritic cells in recognition of hepatitis C virus-infected cells and induction of interferon-alpha. *Hepatology* 58 940–949. 10.1002/hep.25827 22577054 PMC4511847

