# Supplementary figures and images for: Sensing of Porcine Reproductive and Respiratory Syndrome Virus-Infected Macrophages by Plasmacytoid Dendritic Cells
Source: Front Microbiol. 2016 Jun 2;7:771. doi: 10.3389/fmicb.2016.00771 (PMC4937788; doi:10.3389/fmicb.2016.00771)

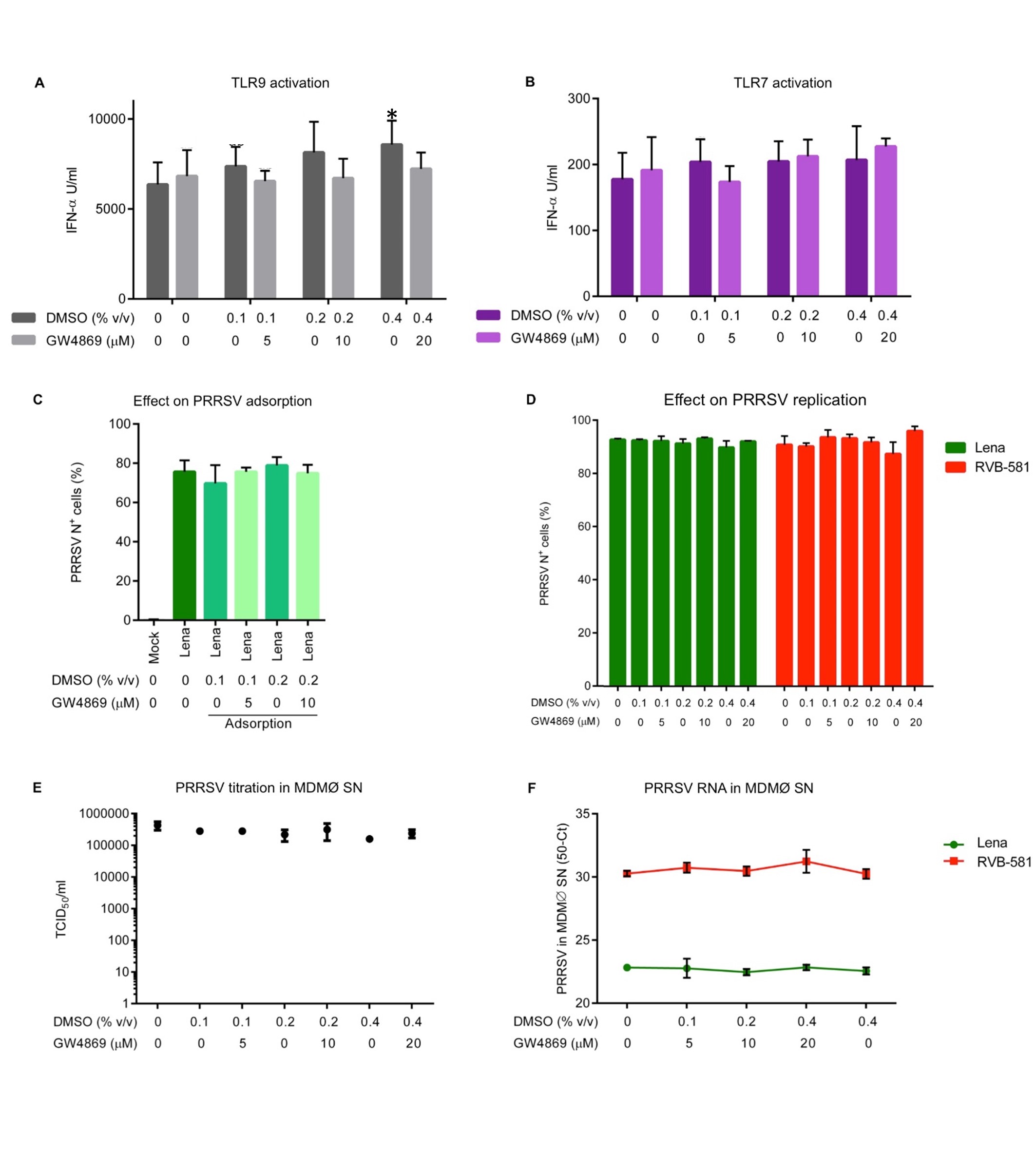

Supplement: Supplementary file 1 [file Image_1.JPG]
